# Occurrence of Black Aspergilli and Ochratoxin A on Grapes in Italy

**DOI:** 10.3390/toxins2040840

**Published:** 2010-04-21

**Authors:** Gianluca Lucchetta, Irene Bazzo, Gianluca Dal Cortivo, Lisa Stringher, Diego Bellotto, Michele Borgo, Elisa Angelini

**Affiliations:** CRA-VIT Centro di Ricerca per la Viticoltura, Viale XXVIII aprile 26, I-31015 Conegliano (TV), Italy; Email: gianluca.lucchetta@gmail.com (G.L.); irene.bazzo@entecra.it (I.B.); gianlucadalcortivo@virgilio.it (G.D.C.); lisa.stringher@email.it (L.S.); diego_bellotto@libero.it (D.B.); michele.borgo@entecra.it (M.B.)

**Keywords:** *Aspergillus* section *Circumdati*, *Aspergillus* section *Nigri*, grapevine, ochratoxin A

## Abstract

Ochratoxin A (OTA) in wine is linked to contamination by several *Aspergillus* species. In 2003–2007, grape samples collected in Italy were surveyed for the presence of OTA and OTA-producing fungi. *A. niger* aggregate was the prevalent species. *A. carbonarius*, which is considered the main source of OTA in grapes, was mostly found in Southern Italy. The year and the environment had an important influence on the development of the black *Aspergillus* populations. Testing with ELISA showed OTA to be present in about 30% of the samples. Samples from Southern Italy showed the highest occurrence (45%) and also the highest OTA concentration, sometimes higher than 2 μg/L. The values decreased progressively the further North the samples were taken.

## 1. Introduction

Ochratoxin A (OTA) is a mycotoxin with immunosuppressive, genotoxic, carcinogenic and potently nephrotoxic properties [1]. Several nephropathies affecting animals and humans have been attributed to OTA; for instance, OTA has been proposed to be associated with the Balkan endemic nephropathy [2]. Based on the available scientific data, the International Agency for Research on Cancer has classified it as a possible human carcinogen in the group 2B [3]. 

OTA occurs naturally in several foodstuffs, including grapes and their derivatives. The first reports of OTA in wine go back to 1996 [4,5]. Since then, many studies have focused on OTA occurrence in products derived from grapes, such as dried vine fruit, wine, grape juice, must and vinegar [6,7]. OTA has been found in wines in many European countries, as well as in America, Asia, Africa and Australia [8]. Red wines have been reported to be contaminated more frequently than white wines, probably due to the different wine-making methods involved [9]. OTA occurrence seems to be higher in wines from Southern European countries: several studies have shown an increase in the amount of OTA in warmer climates [6,10]. Given the danger posed by OTA, the European Community has recently established a toxin concentration limit of 2 μg/kg in grape juice, must, wine and dried fruit (Commission Regulation n. 123/2005/EC). 

OTA is a secondary fungal metabolite produced naturally by several *Aspergillus* and *Penicillium* species; *Aspergillus* and *Penicillum* species able to produce OTA occur in temperate and cold climate areas, respectively. The most important OTA-producing species belong to *Aspergillus* sections *Circumdati* and *Nigri* [11,12]; however, the presence of OTA in grapes and wine is mainly linked to the contamination in the vineyard by species belonging to the *Aspergillus* section *Nigri*, the so-called black aspergilli. The major producer of OTA in grapes is *A. carbonarius*, though other species belonging to the *Nigri* and *Circumdati* sections have also been found to produce the toxin in different Mediterranean countries, such as Spain, Italy, Portugal, and in Australia and South America [13,14,15,16,17,18]. The percentage of *A. carbonarius* strains able to produce OTA and isolated from grapes has been found to range between 70 and 100% when grown *in vitro*, whereas the range of OTA positive strains has been reported to be around 2–20% for *A. niger* and *A. tubingensis* [15,19]. Some reports claimed the production of OTA also by *A. japonicus*, however it is unconfirmed [20,21]. Ponsone *et al*. [22] have recently found that *A. niger* aggregate was the most frequent species on grapes in Argentinean vineyards, with 27% of the isolates producing OTA. The authors also reported the production of OTA by *A. japonicus* and *A. aculeatus* strains; however, that work lacks molecular identification of the strains [23]. Also *A. ochraceus* and other similar species in the section *Circumdati*, in particular *A. westerdjikiae*, have been reported as OTA-producing fungal species in grapes [12,24].

Black aspergilli can be present on grapes from the first stages of berry development and their occurrence increases as the season advances [25]. Meteorological parameters are, indeed, the most important factors in determining the contamination by the black aspergilli, in particular *A. carbonarius* [26]. However, heavy contamination of grapes by OTA-producing species does not necessarily lead to a higher amount of the toxin [27], first of all because not all the strains have the ability to produce the toxin and, secondly, because the production of OTA by these species is influenced by environmental factors, such as humidity and temperature [19].

The aim of this paper was to estimate the occurrence of OTA in grapes from different Italian vine-growing environments and to evaluate the possible correlation with the presence of OTA-producing fungi in the vineyard.

## 2. Results and Discussion

Ochratoxin A (OTA) and fungal contamination were estimated in collected grape samples and the values obtained were analyzed in comparison to the geographic origin of the samples and the climatic data. Moreover, statistical analyses were carried out in order to evaluate whether significant correlations occurred between the following: OTA content and fungal contamination; OTA content and climatic data; fungal contamination and climatic data. 

### 2.1. Ochratoxin A Content

In general, OTA was present in 30.4% of the grape samples analyzed (Table 1). The different geographic origins were responsible for considerable differences in statistical significance. Indeed, the highest number of OTA-contaminated samples came for Southern Italy, where in the five-year period the toxin was detected in 45% of the samples examined. This result was significantly different from data obtained for the samples which were collected in the other regions (p < 0.05). Similar contamination levels have also been recorded in other vineyards in Southern Italy by other authors, in both grapes and wine [10,28,29,30,31]. The lowest occurrence of OTA was recorded in Central Italy, where only 3.3% of the grape samples were contaminated. In Northern Italy, the mycotoxin was present in 17.5% of the tested samples. The difference between the OTA occurrence in Central and Northern areas was not statistically significant. Low occurrence of OTA in samples collected in Northern Italy has been found in previous studies carried out in Piedmont and other regions [29,32].

**Table 1:** The number of OTA-contaminated samples out of the total number of samples collected from the three geographic areas in the different years. Values with different letters are significantly different to each other with the Chi-square analysis (p < 0.05).

Geographic origin	2003	2004	2005	2006	2007	Total
**Northern Italy**	3/22c	1/12c	0/0c	2/19c	5/10c	11/63c
**Central Italy**	1/9c	0/9c	0/0c	0/9c	0/3c	1/30c
**Southern Italy**	20/22a	12/30c	9/26c	4/22c	5/11b	50/111b
**Total**	24/53	13/51	9/26	6/50	10/24	62/204

Moreover, the OTA concentration in the contaminated samples differed according to the origin of the grapes. The highest concentrations of OTA were found in grapes from Southern Italy, with values higher than 2.0 μg/L; one sample showed an OTA concentration as high as 9.2 μg/L. The OTA-contaminated samples from Northern and Central areas did not contain more than 0.02 μg/L of the toxin (Table 2). The climatic differences, related to the geographic region and the latitude, have been demonstrated to influence fungal and OTA contamination: the greatest OTA occurrence and concentration have been found at the lower latitudes [5,10,33,34,35]. 

The OTA occurrence varied greatly according to the year. In general, a higher number of samples were contaminated in 2003 (45.3%) and 2007 (41.7%), while in 2006 the occurrence of OTA-contaminated grapes was as low as 12.0% (Table 1). The highest occurrence of samples contaminated by OTA was recorded in 2003 in grapes from Southern Italy (90.9%). Chi-square analysis highlighted that the latter samples were significantly more contaminated than those collected in the other years and regions (p < 0.05). Differences in OTA levels, probably due to different weather conditions, have been also reported by Pietri *et al*. [10] and Lopez de Cerain *et al*. [36] between samples collected in the same regions in 1995–97 and 1997–98, respectively. 

**Table 2:** OTA concentration range (μg/L) in grape samples collected in Northern, Central and Southern Italy in 2003–2007.

OTA Concentration (μg/L)	Number of Samples			
	Northern Italy	Central Italy	Southern Italy	Total
<0.003	52	29	61	142
0.003–0.020	11	1	23	35
0.02–0.20	0	0	16	16
0.20–2.00	0	0	6	6
>2.00	0	0	5	5
**Total**	63	30	111	204

In the present work, no differences were found between white and red grapes regarding the concentration of the toxin (p > 0.05). On the other hand, analyses carried out on wines instead of grapes have revealed a higher amount of OTA in red wines coming from Tuscany and Sicily in 2000 [37] and from 19 different Italian regions in 1995–97, particularly in wines from Central and Southern Italy [10]. As OTA synthesis during winemaking has not been observed, because alcohol inhibits fungal growth [38], the concentration of the toxin could be higher in red wines due to the presence of the skins during winemaking. In particular, maceration, which is carried out only in red wines, can cause an increase in OTA content estimated at around 20% [21], while fermentation seems to be the primary step responsible for the removal of the toxin [9,39]. Moreover, Caridi *et al*. [40], Bejaoui *et al*. [41] and Leong *et al*. [42] have recently shown that both dead and live yeast cells are able to adsorb OTA rapidly *in vitro*.

### 2.2. Mycological Analyses

**Figure 1:**
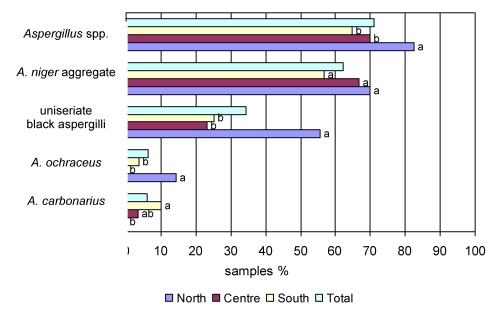
Percentage of grape samples contaminated by different *Aspergillus* species in samples coming from the different geographical areas and overall. Values of the bars labelled with different letters are significantly different to each other with the Chi-square analysis (p < 0.05).

In general, the species belonging to the *Aspergillus* genus (mainly *Aspergillus* section *Nigri* and, sporadically, *A. ochraceus*, belonging to the section *Circumdati*) occurred in more than 70% of the grapes tested. The percentage of contaminated samples varied from one geographic region to another and ranged from 82.5% in the samples from Northern Italy to 64.8% in the grapes from the Southern areas (Figure 1). In contrast, other Italian authors reported that the highest contamination levels by all *Aspergillus* species occurred in grapes from Apulia (Southern Italy) [28,29,30]. The *A. niger* aggregate was the main *Aspergillus* section *Nigri* group of species present in all the samples, with similar occurrence (from 56.8 to 69.8%) in all the different regions; this was also confirmed by the Chi-square test (p > 0.05). The species that were least present in the grape samples were *A. ochraceus* and *A. carbonarius,* which occurred in 0–14.3% and 0–9.9% of the samples, respectively. The occurrence of uniseriate species showed intermediate values, ranging between 23.3% and 55.6%. Black aspergilli species which were different from *A. carbonarius-*mainly *A. niger* aggregate and uniseriate isolates - were present in all the regions surveyed (Figure 1). 

*A. carbonarius*, which is considered the main source of OTA production in grapes, was mainly present in Southern grapes; indeed, it was found in 9.9% of the Southern samples collected in the five-year period. The Chi-square analyses confirmed that grapes from Southern vineyards were significantly more contaminated by *A. carbonarius* than grapes from Northern vineyards. On the contrary, this species was never found in the Northern grapes examined. In Central Italy the occurrence of *A. carbonarius* was intermediate and not significantly different from the occurrence recorded in both Northern and Southern samples (p > 0.05). This geographical distribution of *A. carbonarius* agrees with data reported by other authors in the Mediterranean areas [19]. Battilani *et al.* [29] detected *A*. *carbonarius* in several Northern and Southern Italian regions in the period 2001–2003 and found out that the grape samples from Apulia were the most contaminated by this species, accordingly to the results reported in the present paper.

The strains belonging to the section *Circumdati* were identified as *A. ochraceus* because of their morphological features and the ability to growth at 37 °C: these characteristics were taken into account to distinguish *A. ochraceus* from the quite similar *A. westerdijkiae* [12]. *A. ochraceus*, which usually occurs in much warmer regions than Italy, such as tropical areas, was sporadically found in grapes from both Northern and Southern Italy, in 2003 and 2005, respectively. Using the Chi-square test, the difference in the occurrence of *A. ochraceus* between Northern Italy and the other areas was found to be statistically significant, while no statistical differences between Central and Southern Italy were observed. Although the occurrence of *A. niger* aggregate and *A. carbonarius* is generally higher than that of *A. ochraceus* on grapes [43,44], some authors have detected a higher percentage of OTA positive isolates among *A. ochraceus* in Argentina, Brazil and Spain [24,44]. Therefore, this species should be regarded as a possible contributor to the OTA presence in grapes and their derivatives.

The highest occurrence of uniseriate black aspergilli was in Northern Italy, where 55.6% of the samples were contaminated by these species. The value was statistically different from that recorded in samples collected from Central and Southern Italy, where the occurrence was much lower, with average contamination being 23.3% and 25.2%, respectively. In any case, it has not yet been confirmed that the uniseriate black aspergilli are able to produce OTA.

The percentage of contaminated samples varied greatly from year to year, ranging from 94.3% in 2003 to 36.7% in 2004. In general, the highest occurrence of the *Aspergillus* species was observed in 2003 and 2007, according to the Chi-square analyses (p < 0.05), although there were some differences among the geographical areas. In 2003 and 2005, the highest number of fungal isolates was obtained from the Southern samples, while in 2006 and 2007 it was obtained from the Northern samples. 

### 2.3. Correlation between the Occurrence of OTA and Black Aspergilli

In general, there was no clear correlation between the presence of *A. carbonarius*, uniseriate black aspergillli species, *A. niger* aggregate species and *A. ochraceus* on the one hand and the occurrence and concentration of OTA on the other. In the Northern and Central regions especially, the presence of high populations of these species did not necessarily lead to the production of OTA. However, all the samples from Southern Italy that showed the presence of OTA-producing *Aspergillus* species were contaminated by OTA. 

Some interesting information is provided by samples collected in Apulia in 2003, *i.e.,* the region and the year with the highest production of OTA. The concentration of the toxin was compared with the CFU/g of the different species of *Aspergillus* present in the grape samples (Table 3). The following correlations were statistically significant: *A. niger* aggregate contamination *versus* OTA concentration; *A. carbonarius* contamination *versus* OTA concentration; *Aspergillus* species contamination *versus* OTA concentration. No significant correlation was found in the other years or in the other geographical areas.

**Table 3:** Correlation between the contamination level of different *Aspergillus* species (CFU/g) and the OTA concentration (μg/L) in grapes collected in 2003 in Apulia. ns: not significant; **: p < 0.01; ***: p < 0.001.

Species	r^2^	p	Statistical Significance
*A. carbonarius*	0.3180	0.0063	**
uniseriate black aspergilli	0.0670	0.2690	ns
*A. niger* aggregate	0.5610	0.0010	***
*A. ochraceus*	-	-	-
Total	0.4538	0.0006	***

### 2.4. The Effect of Climatic Conditions on the Occurrence of Black Aspergilli and OTA

**Figure 2:**
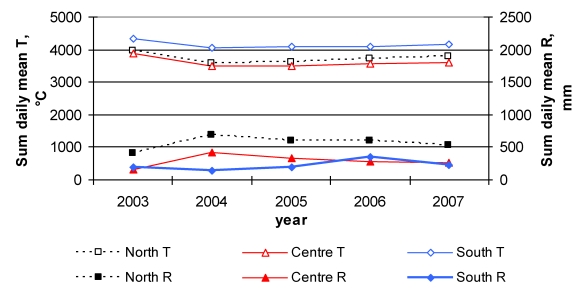
Climatic parameters recorded in three meteorological stations located close to the surveyed vineyards in Northern, Central and Southern Italy. Sum of daily mean temperature (T; °C) and daily rainfall (R; mm) from 1st April to 30th September in the five years of study.

Meteorological conditions, expressed as mean daily temperatures and daily rainfall from 1st April to 30th September were different in the three geographic areas and in the five years of the study (Figure 2). The hottest year was 2003, when the mean daily air temperature was 1,5 °C higher than in 2004 and 2005 and about 1 °C higher than in 2006 and 2007, taking into account the average values for the three areas. It was also dry. The wettest year in Northern and Central Italy was 2004, which was the driest year in Southern Italy (whose wettest year was 2006). 

The temperature trend was similar for Northern, Central and Southern regions in the five-year period; the temperature was very similar both in the North and Center, whilst the Southern areas were much hotter. The rainfall pattern was similar for Northern and Central Italy, although the North was wetter. In Southern Italy, the rainfall pattern was quite different, although the total amount of rain was similar to that in Central Italy. 

It was previously determined by many authors that the highest contamination by OTA-producing fungi occurred on grape samples taken from Southern Italy, where temperatures were higher and humidity was lower. For this reason, more detailed statistical analyses and correlation with meteorological variables were carried out on these samples. Using the Pearson correlation coefficients, an assessment was made regarding the statistical significance of the correlation between the sum of the environmental parameters (maximum, mean and minimum temperatures; maximum, mean and minimum relative humidity; rainfall) in the month before the sampling data and the CFU/g of *A. carbonarius,* uniseriate isolates, *A. niger* aggregate and *Aspergillus* section *Nigri.* The analyses of all the data collected in 2003–2007 revealed a slightly negative correlation only between both *A. niger* aggregate isolates and *Aspergillus* section *Nigri* isolates on the one hand and the minimum and mean relative humidity on the other (p < 0.05). No significant correlation was found with the others parameters and species (Table 4). 

**Table 4:** Correlation values obtained in the comparison between meteorological parameters recorded the month before the sampling and black aspergilli isolated from Apulian grapes, by using the Pearson coefficients. *: statistically significant (p < 0.05).

Meteorological Parameter	*A. carbonarius*	uniseriate black aspergilli	*A. niger* aggregate	*Aspergillus* section *Nigri*
Sum of Maximum Temperature	0.18	0.06	0.16	0.15
Sum of Minimum Temperature	0.18	0.09	0.15	0.15
Sum of Mean Temperature	0.17	0.08	0.13	0.14
Sum of Maximum Relative Humidity	-0.01	0.03	-0.19	-0.15
Sum of Minimun Relative Humidity	-0.10	0.04	-0.26*	-0.21*
Sum of Mean Relative Humidity	-0.07	0.02	-0.26*	-0.21*
Sum of Rainfall	0.08	0.13	-0.02	0.02

The data were confirmed using the daily average of each parameter and the CFU/g of *Aspergillus* section *Nigri* (data not shown)*.* This result highlighted that the grapes were more contaminated by these OTA-producing fungi in the years when it was very dry. Other authors have also demonstrated that the meteorological conditions play a major role both in fungal colonization in bunches: the year significantly affected the number of berries colonized by black aspergilli, particularly *A. carbonarius* at harvesting, with a positive correlation with the sum of degree-day and negative with the sum of rain between early veraison and ripening [29]. In a different paper, a positive correlation has been observed between temperature and grape contamination by black aspergilli, while correlation with relative humidity and rainfalls has not always been evident [45]. 

Moreover, OTA was mainly found in the hottest and driest years and areas. Indeed, in 2003, which was the hottest and driest year, a significantly higher number of samples contaminated by OTA was found (p < 0.05). Meteorological conditions, as well as closeness to the sea, have been shown to play a major role in determining OTA occurrence in grapes [9].

## 3. Experimental Section

### 3.1. Grape Samples

Grapes were collected in 23 vineyards located in four Italian regions: Veneto (North), Tuscany and Latium (Center) and Apulia (South), during the 2003–2007 harvests. Twenty-two different grapevine varieties were included, among which 16 were wine varieties (11 red and 5 white) and six table grape varieties (3 red and 3 white) (Table 5).

Sampling was carried out in a systematic manner: each row tested was divided into three parts (beginning, middle and final), which were used as replicates. In every replicate, about 50 berry clusters were randomly collected from different parts of the bunch and from different positions on the plant, in order to obtain a representative sample. In total, 204 samples (800 to 1000 g) were collected in plastic bags and maintained in refrigerated containers until they were processed (maximum 2 days). Each sample was hand-crushed and homogenized in a plastic bag and then divided into two aliquots: one for OTA detection and another for the evaluation of fungal contamination. The aliquot for OTA detection was maintained at -20 °C until the analyses, whilst the aliquot for the evaluation of fungal contamination was immediately processed.

**Table 5:** Features of grapevine samples collected for the evaluation of OTA and mycological contamination.

Region	Province	Grapevine variety	Berry color	Use	Number of samples
Latium	Frosinone	Bellone	white	wine	1
Latium	Frosinone	Malvasia di Candia	white	wine	1
Latium	Roma	Malvasia di Candia	white	wine	2
Apulia	Bari	Aglianico	red	wine	4
Apulia	Bari	Italia	white	table	7
Apulia	Lecce	Negramaro	red	wine	1
Apulia	Bari	Merlot	red	wine	2
Apulia	Bari	Montepulciano	red	wine	15
Apulia	Bari	Negramaro	red	wine	5
Apulia	Bari	Primitivo	red	wine	12
Apulia	Bari	Primitivo	red	wine	6
Apulia	Taranto	Italia	white	table	2
Apulia	Taranto	Crimson	red	table	1
Apulia	Taranto	Italia	white	table	1
Apulia	Taranto	Regina	white	table	2
Apulia	Bari	M. Palieri	red	table	2
Apulia	Bari	Victoria	white	table	3
Apulia	Taranto	Italia	white	table	2
Apulia	Taranto	Italia	white	table	3
Apulia	Taranto	Red Globe	red	table	1
Apulia	Taranto	Crimson	red	table	1
Apulia	Brindisi	Cabernet Sauvignon	red	wine	4
Apulia	Brindisi	Malvasia nera	red	wine	4
Apulia	Brindisi	Merlot	red	wine	4
Apulia	Brindisi	Montepulciano	red	wine	4
Apulia	Brindisi	Negramaro	red	wine	4
Apulia	Brindisi	Primitivo	red	wine	4
Apulia	Bari	Cabernet Sauvignon	red	wine	2
Apulia	Bari	M. Palieri	red	table	3
Apulia	Bari	Malvasia	white	wine	2
Apulia	Bari	Merlot	red	wine	2
Apulia	Bari	Montepulciano	red	wine	2
Apulia	Bari	Negramaro	red	wine	2
Apulia	Bari	Primitivo	red	wine	2
Apulia	Bari	Primitivo	red	wine	2
Tuscany	Siena	Sangiovese	red	wine	4
Tuscany	Arezzo	Merlot	red	wine	3
Tuscany	Arezzo	Montepulciano	red	wine	6
Tuscany	Arezzo	Sangiovese	red	wine	13
Veneto	Treviso	Raboso piave	red	wine	3
Veneto	Treviso	Nero d'Avola	red	wine	1
Veneto	Verona	Merlot	red	wine	2
Veneto	Treviso	Chardonnay	white	wine	3
Veneto	Treviso	Merlot	red	wine	36
Veneto	Treviso	Negramaro	red	wine	5
Veneto	Treviso	Pinot grigio	white	wine	3
Veneto	Treviso	Primitivo	red	wine	7
Veneto	Treviso	Sangiovese	red	wine	2
Veneto	Treviso	Fiano	white	wine	1
**Total samples**					**204**

### 3.2. Ochratoxin A Extraction and Determination

The protocol for OTA determination in the grape samples included an extraction of the toxin using immunoaffinity columns (IAC) and immunoassay by competitive enzyme-linked immunosorbent assay (cELISA), in accordance with Angelini *et al*. [46]. In order to remove any solid residue, samples were subjected to a gross filtration and centrifugation stage of 15 min at 2000 × g; 100 mL of the supernatant was then filtered with glass micro-fiber filters (Whatman, grade GF/A) under vacuum and stored at -20 °C until analysis.

OTA was extracted using RIDA Ochratoxin A columns (R-Biopharm), following the manufacturer's instructions for wine, with minor alterations. Ten milliliters of the clarified sample were diluted twice with a sodium phosphate buffer (0.4 M, pH 7.5), following which 10 mL of the solution obtained was applied directly to the IA columns. The columns were washed with a 9:1 solution of sodium phosphate buffer (PBS 20 mM, pH 7.4) and methanol, and dried by air flushing. Any toxin present was subsequently eluted with methanol, evaporated at 40 °C overnight and then prepared for the cELISA. The dried pellets obtained from the IAC extractions were stored at -20 °C in the dark and reconstituted with 0.5 mL of a sodium bicarbonate buffer (0.13 M, pH 8.1) immediately prior to immunoassay.

Immunoassays were carried out using commercial kits (R-Biopharm), suitable for OTA determination in different foods and feeds. The cELISA kit included a 96-well microplate, six OTA standards, an anti-OTA antibody and an enzymatic conjugate, developing and stop solutions and a washing buffer. Blank, OTA standard and sample wells were always run in duplicate. The enzyme immunoassay was performed following protocols provided by the manufacturer, with incubation taking place in the dark at room temperature (20–25 °C). Absorbance was measured after the last incubation step at an optical density of 450 nm in a spectrophotometer (Titertek Multiscan Plus MKII, Labsystem). Whenever the absorbance was higher than the upper limit of detection, samples were diluted and analyzed again.

The analyses of the cELISA data were performed using the calibration curves, which were constructed with five points. The curve for each plate was obtained from the mean absorbance values of each OTA standard included in the kit. The values of the toxin concentration in the samples were calculated from the mean absorbance values by interpolating the corresponding OTA concentrations from the calibration curve. The software used for the analysis of the results was RIDA®SOFT Win (R-Biopharm), which was provided by the manufacturer.

### 3.3. Mycological Analyses

The mycological analyses of the grape samples were performed using a serial dilution plating method. Two-hundred grams of juice and 200 g of pulp, skins and stalks were placed in a sterile flask and diluted twice with a sterile 0.1% bacteriological peptone solution (Oxoid), before shaking on an orbital shaker for 15 min to allow adequate suspension of the fungal conidia, spores and fragments of mycelium. Finally, 50 mL of each sample were collected and five ten-fold serial dilutions from 1:1 to 1:10000 were performed. One-hundred microliters of each dilution was poured onto three Petri dishes and spread with a sterilized glass rod over the whole substrate surface, until fully absorbed. The culture medium used was Dichloran Yeast Extract Sucrose 18% Glycerol agar (DYSG), amended by chloramphenicol, in accordance with Pitt and Hocking [47]. The Petri dishes were placed in plastic bags and incubated in the dark for 6–7 days in a climatic cabinet at 25 °C, until colonies formed. The fungal colonies were then counted and the strains belonging to the *Aspergillus* genus were isolated on Czapek Yeast extract Agar (CYA) medium for identification at species level.

The identification focused on species belonging to the *Nigri* and *Circumdati* sections, which are OTA-producers, although all isolates belonging to the *Aspergillus* genus were isolated and identified. The identification at species level was carried out on the basis of the macroscopic and microscopic features of the fungal isolates. The following were recorded on different substrates and at different temperatures: the growth diameter of the colonies; the characteristics and color of the mycelium; the production of pigments and exudates; the characteristics of the conidiophores and conidia [47,48]. Microscopic observation of reproductive structures from colonies grown in MEA (Malt Extract Agar) was performed by glass slide preparation in absolute ethanol and lactic acid and observation at 400 and 1000 X magnification with an optical microscope (Leitz Laborlux S). *Aspergillus* section *Nigri* isolates were identified as uniseriates, biseriates (mainly isolates belonging to the *A.* *niger* aggregate) or *A. carbonarius*. Uniseriate isolates bear uniseriate conidial heads, while biseriates bear biseriate heads; among the latter, *A. carbonarius* isolates were identified at species level, whilst the other biseriate isolates were, on the whole, classified as *A.* *niger* aggregate. When present, isolates of the section *Circumdati* were identified at species level [12,47,48,49]. 

For every species, the amount of Colony Forming Units per gram of fresh grape weight (CFU/g) was evaluated. The strains were preserved for further analyses and characterization by taking pieces of mycelium and substrate and placing them at -80 °C in tubes with sterile 10% glycerol solution.

### 3.4. Meteorological and Climatic Data

Climatic data were gathered from meteorological stations located close to the vineyards from which the samples were collected. In Northern Italy, the data were obtained from the meteorological station of “C.R.A. – Centro di Ricerca per la Viticoltura”, Conegliano (TV); in Central Italy, the climatic data were kindly furnished by M. D’Arcangelo (C.R.A. – Unità di Ricerca per la Viticoltura, Arezzo); in Southern Italy, the climatic data were obtained from the meteorological station of “Associazione Consorzi di Difesa della Puglia". Rainfall, maximum, mean and minimum daily air temperature and relative humidity were recorded and used in the subsequent statistical correlation analyses with the occurrence of *Aspergillus* species section *Nigri* and *Circumdati* and OTA.

### 3.5. Statistical Analyses

Several analyses were carried out in order to check whether there was any statistically significant correlation between OTA content, fungal contamination, geographic areas and climatic conditions. The Pearson correlation coefficients, the Chi-square analysis and the linear regression (r^2^) were carried out using Statistica 7.1 software (StatSoft, Inc. Tulsa, OK).

## 4. Conclusions

The occurrence of OTA and OTA-producing fungi in Italian grapes and in grapes in other vine-growing countries has been reported by several studies, most of which have been carried out in the last few years. The results of the present work confirmed the high risk of OTA and fungal contamination in Southern Italy, where in very dry and hot years, such as demonstrated for 2003, OTA concentration on grapes can reach very high levels. On the other hand, the presence of OTA-producing fungi in the Central and Northern regions did not lead to the production of the toxin at a level which is dangerous for human health in the years studied. On the whole, OTA concentration levels over the legal limit were found in 2.5% of more than 200 grape samples tested over five years.

OTA is a problem that originates in the vineyard. Black aspergilli, the main fungi responsible for OTA presence in grapes, are naturally present in vineyards, and fungi can be isolated from bunches starting from the early stages of the berry development, although their incidence is more relevant from early veraison. Despite the widespread occurrence of OTA in various types of wine, there is limited information on the ability of black aspergilli to infect berries and produce OTA in different grape varieties. The ecological parameters of black aspergilli are not completely known and this knowledge is critical in the development and prediction of the risk models on contamination of grapes [9].

Climatic conditions and geographical location are important factors favoring OTA accumulation in grape berries. Damaged berries, by abiotic or biotic causes, provide preferential entries for black aspergilli and their efficiency in producing OTA increases [50]. High OTA levels occur in grapes severely damaged by the grape moth, *Lobesia botrana*, particularly in the Mediterranean areas [9,51]. 

Control measures for toxigenic mycoflora in the vineyards must consider these critical control points. Moreover, it is necessary to monitor OTA in grapes and their derivatives products, especially in areas with the highest risk of occurrence of the toxin and the OTA-producing fungi.
